# Clinical outcomes of different implant types in mandibular bar-retained overdentures: a retrospective analysis with up to 20 years follow-up

**DOI:** 10.1186/s40729-022-00439-x

**Published:** 2022-09-23

**Authors:** M. Betthäuser, R. Schilter, N. Enkling, V. G. A. Suter, S. Abou-Ayash, M. Schimmel

**Affiliations:** 1 Department of Reconstructive Dentistry and Gerodontology, School of Dental Medicine, University of Bern, Bern, Switzerland; 2 Department of Prosthodontics, Preclinical Education and Dental Materials Science, Medical Faculty, University of Bonn, Bonn, Germany; 3 Department of Oral Surgery and Stomatology, School of Dental Medicine, University of Bern, Bern, Switzerland; 4 Division of Gerodontology and Removable Prosthodontics, University Clinics of Dental Medicine, University of Geneva, Geneva, Switzerland

**Keywords:** Implant overdentures, Bar-attachments, Peri-implant bone-level changes, Implant success, Implant survival

## Abstract

**Purpose:**

To determine the clinical and radiological outcomes of hybrid-design- (HD) and bone-level (BL) implants for bar-retained mandibular implant-overdentures (IODs).

**Methods:**

For this retrospective study, edentulous patients who had received maxillary complete dentures and mandibular bar-retained IODs were invited for a follow-up assessment. Implant survival, implant success and health of peri-implant tissues were assessed on an implant level-based analysis. Patient-based parameters served to identify risk factors for peri-implant bone loss, presence of peri-implantitis and success.

**Results:**

Eighty patients (median age 72.72 [67.03; 78.81] years, 46 females) with 180 implants (median follow-up 12.01 [10.82; 21.04] years) were assessed. There was no difference concerning the rate of implant failure (*p* = 0.26), or peri-implantitis (*p* = 0.97) between HD and BL implants. Solely in one study group, there was the presence of peri-implant pus. Implant success was higher in BL implants with one group being notably higher than the comparing groups (*p* = 0.045). For bone loss, a width of keratinized mucosa (KM) ≤ 1 mm (*p* = 0.0006) and the presence of xerostomia (*p* = 0.09) were identified as risk factors. Smoking (*p* = 0.013) and a higher body mass index (BMI) (*p* = 0.03) were a risk factor for peri-implantitis. As risk factors for reduced implant success, a small width of KM (*p* = 0.003) and the presence of xerostomia (*p* = 0.007) were identified.

**Conclusions:**

For mandibular bar-retained IODs, both BL and HD implants are mostly successful. A minimum of 1 mm KM around implants and normal salivary flow are relevant factors for implant success and stable peri-implant bone levels. Smoking and a high BMI are potential risk factors for peri-implantitis.

## Background

Implant overdentures (IODs) retained by two implants have been defined as the recommended standard of care for the edentulous mandible, due to their superiority compared to mucosa-borne complete dentures in terms of clinical- and patient-reported outcomes [1].

Survival rates of dental implants in edentulous jaws are high, with slightly higher survival rates in fixed compared to removable restorations [2, 3]. Nevertheless, a considerable number of patients experience implant failure with subsequent reduced quality of life and high costs for replacement and denture modification [4]. While early implant failure is often caused by impaired or failed osseointegration, late implant failure occurs when osseointegration cannot be maintained. Often, inflammation of the peri-implant tissues (peri-implant mucositis or peri-implantitis) is a causal factor. While peri-implant mucositis—if treated early—may resolve without sequelae, progression of the disease usually involves osseous structures (i.e., peri-implantitis), thus increasing the risk of failure with subsequent implant loss [5]. Today, there is very little scientific evidence on the peri-implant health of dental implants supporting overdentures [6].

The macro-design of endosseous dental implants is extremely variable, but can be broadly described as bone-level (BL), or tissue-level with machined neck surfaces in various configurations. Depending on the implant manufacturer, tissue-level type implants are also called hybrid-design (HD) implants [7]. Furthermore, there is a multitude of implant surfaces from machined surfaces to various surface modifications, such as the SLA®, SLAactive®, TiUnite® or the SICMatrix® surface.

Derks et al. reported in their population-based studies, that the implant brand, and therefore the macro- and micro-design as well as the implant surface, might play a role in implant survival and peri-implant health. In their analysis, there is indirect evidence that HD tissue-level implants might perform better in regard to implant failure and peri-implant tissues reaction than BL-implants. However, they did not distinguish between partially or completely edentulous indications [8, 9].

Hence, there is still no conclusive evidence which implant might perform best for the retention of an IOD in terms of implant success and implant survival. Different available implant surface characteristics complicate the decision and there is preliminary evidence that the implant surface might play a role in the development of peri-implant inflammation [10].

The choice of implant brand and design for retaining IODs mostly depend on the preference of the dentist—evidence-based guidelines are missing and manufacturers rarely give recommendations for a specific indication. Therefore, the aim of this retrospective controlled study with clinical and radiological examination was to report mean peri-implant bone-level changes (– ∆MBL) as well as clinical conditions in completely edentulous patients provided with mandibular implant-bar supported IODs. The tested null-hypothesis H0 was: "Edentulous patients with mandibular bar-retained IODs show the same prevalence and severity of peri-implantitis/peri-implant mucositis irrespective of the implant type.”

## Materials and methods

This retrospective controlled cohort study with clinical and radiological assessment was conducted according to the guidelines published as the STROBE statement [11] and was carried out in compliance with Good Clinical Practice and the ethical standards by the current version of the Declaration of Helsinki. The study protocol was approved by the Ethics Committee of Bern, Switzerland (KEK-Nr. 268/15).

Patient records of the Department of Reconstructive Dentistry and Gerodontology, University of Bern, Switzerland were screened systematically. Inclusion criteria were: being fully edentulous, having received a mandibular bar-retained IOD (implants in the interforaminal region) between 1999 and 2015 and having a baseline radiograph. Exclusion criteria were: any condition interfering with the capability of providing written informed consent, pregnancy or lactation, compromising medical conditions like immunosuppressive therapy, history of head and neck radiation or chemotherapy or change of attachment during follow-up.

### Study protocol

The patient record screening resulted in a list of potential study participants. They were contacted by telephone and invited to participate in the present study. After written informed consent was obtained, participants attended a free follow-up assessment providing medical and dental history, clinical and radiological assessment between November 2018 and May 2020.

Two dentists not involved in the initial treatments of the patients performed the clinical assessments. In this context, the first six subjects were evaluated twice by each dentist for calibration purposes according to pre-defined standard examination procedures.

Xerostomia was evaluated with the German version of the Xerostomia Inventory (XI-G). It comprises 14 questions that related to a dry mouth and throat using a 5-point ordinal scale; the sum score ranges from 0 to 56 with higher values indicating more symptoms of dry mouth [12].

**Fig. 1:**
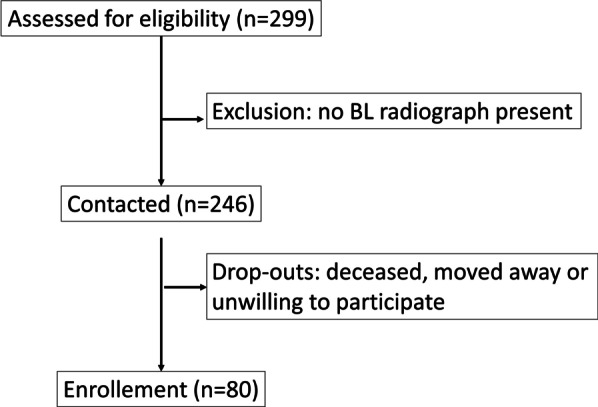
Flowchart of the study enrollment

**Fig. 2:**
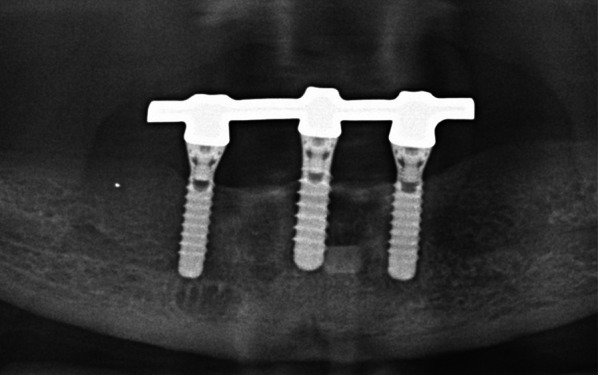
Exemplary radiograph of the implant group HD-SLA (Institut Straumann, Basel, Switzerland)

**Fig. 3:**
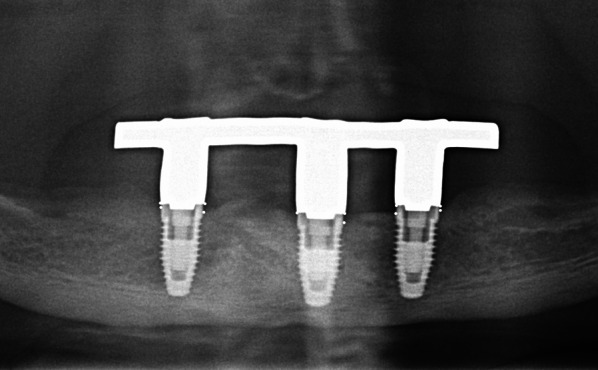
Exemplary radiograph of the implant group HD-TiUnite with machined collar (Nobel Biocare AB, Zurich, Switzerland)

**Fig. 4:**
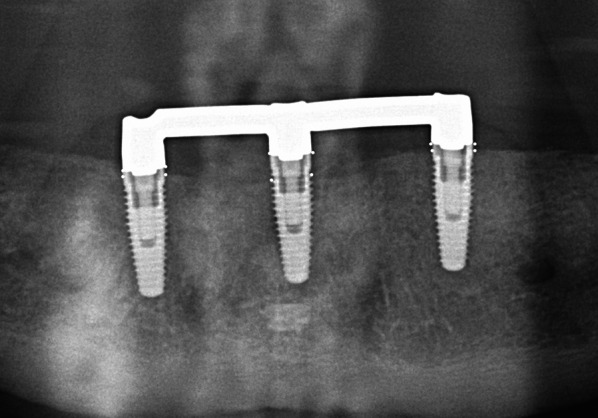
Exemplary radiograph of the implant group BL-TiUnite with surface on collar (Nobel Biocare AB, Zurich, Switzerland)

Participants were grouped according to the type of implant they had received: tissue-level implants with machined neck (“hybrid design implants”, HD) with SLA/SLActive surface which is created by aluminum oxide blasting and acid etching (HD-SLA, Institut Straumann, Basel, Switzerland, Fig. 1), TiUnite surface which is created by anodic oxidation, and machined neck (HD-TiUnite, Nobel Biocare AB, Zurich, Switzerland, Fig. 2), bone-level implants (BL) with TiUnite surface (BL-TiUnite, Nobel Biocare AB, Zurich, Switzerland, Fig. 3), or BL with SICmatrix surface which is created by blasting with round zirconia granules and acid etching (BL-SIC, SICmatrix at the SICace implant; SIC invent AG, Basel, Switzerland, Fig. 4).

### Clinical examination

In the present study, bleeding on probing (BOP, yes/no), and probing depth (PD) using a millimeter-scaled probe (largest value per implant) were assessed at 4 sites per implant (mesial/distal/buccal/oral) [13]. For PD, the difference between initial value at implant loading and study-related assessment was calculated (∆PD (mm)). The keratinized mucosa (KM) was evaluated at 2 sites per implant (buccal/oral) and for statistical analyses, KM was defined as a binary factor, with levels < 1 mm and ≥ 1 mm, based on the smaller of the two measurements at follow-up. Furthermore, the O’Leary Plaque Index on four aspects of each implant were assessed and the mean of the measurements were used for statistical analysis [14].

### Radiological examination

Baseline radiographs from the patient files and the radiographs taken during the study-related examination served to evaluate – ∆MBL. For groups HD-SLA, HD-TiUnite, BL-TiUnite, the assessment was performed on panoramic radiographs, for the group BL-SIC standardized peri-apical radiographs were available both for baseline and follow-up [15].

The software package ImageJ version 1.44 (https://imagej.nih.gov/ij/) was used with 200% digital magnification of the images. All measurements were performed after calibrating the software based on the known length of the thread pitch: 1.25 mm for HD-SLA, 0.64, 0.75, 1.2 mm depending on the implant diameter for HD-TiUnite and BL-TiUnite and 0.8 mm for BL-SIC. The distance between implant shoulder and first bone–implant contact was measured on the mesial and distal aspect. The error of the method was calculated by repeating a sample of 15 measurements per group for calibrating reasons of the investigator (MB).

– ∆MBL was calculated for each implant separately as the maximum difference in distances between implant shoulder and first bone–implant contact from the day of implant placement to date of study-related assessment. Bone-loss is reported as – ∆MBL. The mean of mesial and distal served as – ∆MBL/implant for statistical analysis [16].

### Diagnosis of peri-implantitis and peri-implant mucositis

Peri-implantitis was assessed, based on the criteria described by Berglund et al. (2018). Briefly, the presence of bleeding and/or suppuration on gentle probing, increasing probing depths compared to previous examinations and presence of radiographic bone loss compared to implant placement would qualify for the diagnosis. In the absence of previous examination data, following indices in combinations were applicable criteria: probing depth (PD) of ≥ 6 mm, bone levels ≥ 3 mm apical of the most coronal portion of the intraosseous part of the implant [17]. Its precursor, peri-implant mucositis, is characterized by bleeding on gentle probing and may comprise erythema, swelling or suppuration.

### Implant success and survival

Implant success and survival was assessed according to Misch et al. (2008). An implant was considered a success if grade 1 (optimum health) was diagnosed according to the following criteria: no pain or tenderness upon function, no mobility, < 2 mm radiographic bone loss from initial surgery and no exudates history. An implant was considered unsuccessful with grades 2–4 according to Misch et al. (2008). Satisfactory survival (grade 2) comprises the factors 2–4 mm radiographic – ∆MBL and otherwise no pain or tenderness upon function and no mobility. Compromised survival (grade 3) was defined as – ∆MBL of ≥ 4 mm or clinical outcomes such as no sensitivity on function, PD > 7 mm or an exudates history. Failure (grade 4) comprises the factors pain on function, mobility, a – ∆MBL > ½ implant length, uncontrolled exudate or the implant being lost [18].

### Statistical analysis

Sample size calculation was based on the main outcome parameter “prevalence of peri-implantitis” based on previously published effects sizes [19]. Accordingly, a minimum of n = 30 implants must have been included in every group to achieve an odds ratio (OR) > 1. Descriptive analysis comprised medians [1st quantile; 3rd quantile] for metric and percentages for binary outcomes.

Demographic parameters age, gender (binary), smoking habits (binary), diabetes mellitus type 1/type 2 (binary yes/no), anticoagulants (binary yes/ no), body mass index (BMI), XI-G and recall interval were recorded and assessed patient-wisely while clinical parameters implant loss (binary), presence of pus (binary), BOP (binary), changes in PD (∆PD), plaque index (binary), KM (binary, ≥ 1 mm and < 1 mm), changes in mean bone loss (– ∆MBL), peri-implantitis (binary) and implant success (binary, grade 1 [optimum health], and grade 2 or higher [compromised success] [18]), were recorded and assessed implant-wisely.

Patient- and implant-related outcomes were tested for differences between implant groups HD-SLA, HD-TiUnite, BL-TiUnite, BL-SIC and between implant types (HD vs. BL) using either Mann–Whitney tests or Kruskal–Wallis tests on a global scale with Mann–Whitney tests post hoc for metric outcomes or exact Fisher tests on a global scale (extended version if design larger than 2 × 2) and post hoc for binary outcomes. Furthermore, it was tested whether the parameters age, gender, BMI, smoking habits, diabetes mellitus, anticoagulants, xerostomia, recall interval, KM, plaque index and implant groups have an impact on outcomes – ∆MBL, peri-implantitis and implant success. To assess the impact on – ∆MBL, a univariate screening was first conducted using Mann–Whitney tests and Spearman rank-correlation. All significant parameters from screening with more than n = 150 observations were then carried over to a final multivariate linear regression model. A similar procedure was used for binary outcomes peri-implantitis and implant success, but using logistic regression models this time (for screening and final model). Finally, all final models (linear and logistic) underwent a backward stepwise selection algorithm minimizing the Akaike Information Criterion (AIC), reducing all models to only “risk parameters” for the respective outcome [20]. Goodness-of-fit of final models were assessed by checking for normality of residuals and homoscedasticity (linear regression) and by checking residuals and applying the Hosmer–Lemeshow test (logistic regression). Implant-related outcomes were not corrected for patients, leading to a possible bias in these assessments. Throughout, *p*-values smaller or equal to 0.05 were considered as statistically significant. All *p*-values for post hoc tests were corrected using the method of “Holm”. All analyses in this report were performed with the statistics software R, version 3.5.0 and by a specialist biostatistician (significantis GmbH, Bern, Switzerland).

## Results

After screening the patient records, a total number of 246 eligible subjects were identified (Fig. 1). All of them were contacted by telephone and 80 patients (median age 72.72 [67.03; 78.81] years, 46 females, 34 males) agreed to participate.

**Fig. 5:**
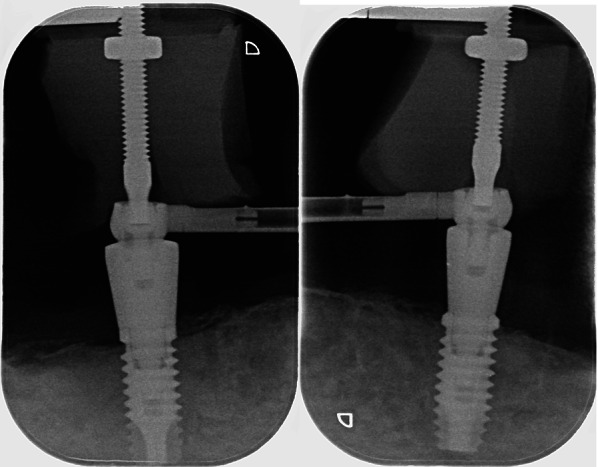
Exemplary radiograph of the implant group BL-SIC (SIC invent AG, Basel, Switzerland)

All participants wore upper complete dentures and mandibular bar-retained IODs supported by 2–4 implants (Figs. 2, 3, 4, 5). In sum, n = 180 implants were assessed. There were 52 implants in the HD-TiUnite group (28.9%), 54 implants in the BL-TiUnite group (30.0%), 52 implants in the HD-SLA group (28.9%) and 22 implants in the BL- SIC group (12.2%). The implants had been in place for a median of 12.01 [10.82; 21.04] years (Table 1).

**Table 1:** Descriptive of implant characteristics, time under function, presence of bleeding on probing and implant loss

		HD-TiUnite n (%)	HD-SLA n (%)	BL-SIC n (%)	BL-TiUnite n (%)
Implant diameter	≤ 4 mm	17 (32.69)	13 (25)	22 (100)	12 (22.22)
	> 4 mm	35 (67.31)	39 (75)	0 (0)	42 (77.78)
Time under function	5–10 years	22 (42.31)	4 (7.69)	4 (18.18)	1 (1.86)
	11–15 years	30 (57.69)	34 (65.38)	18 (81.82)	53 (98.15)
	16–21 years	0 (0)	14 (26.92)	0 (0)	0 (0)
Bleeding on probing	Yes	32 (61.54)	26 (50)	6 (27.27)	16 (29.63)
	No	20 (38.46)	26 (50)	16 (72.73)	38 (70.37)
Loss of implant	No	49 (94.23)	52 (100)	22 (100)	51 (94.44)
	Late loss (≥ 1 year after placement)	0 (0)	0 (0)	0 (0)	2 (3.70)
	Early loss (< 1 year after placement)	3 (5.77)	0 (0)	0 (0)	1 (1.85)

In the sample, there were 15 smokers (18.8%), 10 with diabetes mellitus (12.5%) and 32 taking anticoagulants (40.0%). Median body mass index (BMI was 27.29 [23.84; 30.75] kg/m^2^, 29 missing values) and median XI-G was 10.50 [2.75; 16.00]. The median recall interval was 10.40 [8.40; 12.20] months (Table 2).

**Table 2:** Differences between possible risk factors by implant-level and implant-type

Variable	Total	HD-implants	BL-implants	*p*-value^*^	HD-TiUnite	HD-SLA	BL-TiUnite	BL-SIC	*p*-value^**^
Age	72.72 [67.03, 78.81]	70.92 [66.46, 78.58]	73.97 [69.66, 78.78]	0.40	69.08 [64.40, 76.30]	75.83 [68.95, 79.33]	74.08 [69.89, 82.08]	73.09 [69.23,77.00]	0.18
Smoking Ref: yes	15/80 (18.8%)	5/46 (10.9%)	10/34 (29.4%)	0.30	3/24 (12.5%)	2/22 (9.1%)	7/23 (30.4%)	3/11 (27.3%)	0.21
Diabetes Ref: yes	10/80 (12.5%)	8/46 (17.4%)	2/34 (5.9%)	0.18	3/24 (12.5%)	5/22 (22.7%)	1/23 (4.3%)	1/11 (9.1%)	0.32
Anticoagulants Ref: yes	32/80 (40.0%)	20/46 (43.5%)	12/34 (35.3%)	0.50	11/24 (45.9%)	9/22 (40.9%)	8/23 (34.8%)	4/11 (36.4%)	0.89
BMI	27.29 [23.84, 30.75]	27.34 [24.39, 31.14]	25.99 [22.95, 28.08]	0.20	29.09 [25.56, 31.64]	26.28 [23.12, 28.04]	25.99 [23.43, 27.85]	24.52 [22.64, 26.40]	0.24
Xerostomia	10.50 [2.75, 16.00]	12.00 [4.25, 16.00]	6.00 [1.25, 14.75]	0.08	13.00 [6.00, 18.00]	11.50 [4.25, 14.00]	3.00 [0.50,18.00]	9.00 [4.00, 12.00]	0.33
Recall interval (m)	10.40 [8.40, 12.20]	10.45 [8.43, 12.20]	10.40 [8.40, 12.10]	0.93	10.00 [7.73, 11.75]	11.25 [9.83, 12.75]	8.85^a^ [7.50, 10.70]	11.70^a^ [10.90, 18.55]	0.005
Gender Ref: female	46/80 (57.5%)	29/46 (63.0%)	17/34 (50.0%)	0.26	14/24 (58.3%)	15/22 (68.2%)	13/23 (56.5%)	4/11 (36.4)	0.40

^*^P-value comparing HD- with BL-implants, ^**^P-value comparing implant types. ^a^Groups with same letter show statistically significant post hoc tests

### Radiological and clinical outcomes

Median – ∆MBL was 1.57 mm [0.69, 2.68] and median ∆PD was 2.00 mm [1.41, 2.50]. 35 patients suffered from peri-implantitis (19.7%), 102 had a positive BOP (56.7%). Implant success was recorded in 107 patients (60.1%) (Table 3).

**Table 3:** Differences of tested parameters and possible risk factor by implant-level and implant-type

Variable	Total	HD-implants	BL-implants	*p*-value^*^	HD-TiUnite	HD-SLA	BL-TiUnite	BL-SIC	*p*-value^**^
Loss of implant Ref: yes	6/180 (3.3%)	3/104 (2.9%)	3/76 (3.9%)	0.70	3/52 (5.8%)	0/52 (0.0%)	3/54 (5.6%)	0/22 (0.0%)	0.26
BOP Ref: yes	102/180 (56.7%)	58/104 (55.8%)	44/76 (57.9%)	0.88	32/52 (61.5%)	26/52 (50.0%)	38/54^a^ (70.4%)	6/22^a^ (27.3%)	0.004
KM Ref: KM ≥ 1 mm	120/176 (68.2%)	73/104 (70.2%)	47/72 (65.3%)	0.51	39/52 (75.0%)	34/52 (65.4%)	31/52 (57.4%)	16/20 (80.0%)	0.24
Peri-implantitis Ref: yes	35/178 (19.7%)	20/104 (19.2%)	15/74 (20.3%)	1.00	11/52 (21.2%)	9/52 (17.3%)	11/52 (21.2%)	4/22 (18.2%)	0.97
PUS Ref: yes	5/178 (2.8%)	0/104 (0.0%)	5/74 (6.8%)	0.01	0/52 (0.0%)	0/52 (0.0%)	5/52 (9.6%)	0/22 (0.0%)	0.01
Plaque index Ref: yes	167/178 (93.8%)	97/104 (93.3%)	70/74 (94.6%)	1.00	49/52 (94.2%)	48/52 (92.3%)	50/52 (96.2%)	20/22 (90.9%)	0.83
ΔPD	2.00 [1.41, 2.50]	2.13 [1.41, 2.50]	1.75 [1.25, 2.75]	0.60	1.69 [1.22, 3.03]	2.25 [1.94, 2.50]	1.75 [1.25, 2.28]	2.25 [1.75,2.94]	0.07
-ΔMBL	1.57 [0.69, 2.68]	1.95 [0.90, 3.02]	1.48 [0.48, 1.96]	0.21	1.83 [0.83, 2.78]	1.82 [0.86,2.91]	1.60 [0.68, 2.61]	1.23 [0.48, 1.90]	0.37
Implant success Ref: optimal health	107/178 (60.1%)	56/104 (53.8%)	51/74 (68.9%)	0.045	26/52 (50.0%)	30/52 (57.7%)	33/52 (63.5%)	18/22 (81.8%)	0.07

^*^P-value comparing HD- with BL-implants, ^**^P-value comparing implant types. ^a^Groups with same letter show statistically significant post hoc tests

### Assessment of implant groups

Implants were also grouped according to their macro-design, HD and BL and group-wisely for both patient- and implant-related parameters (Tables 2, 3). Considering patient-related parameters, no significant differences were found on implant level, although the *p*-value comparing the XI-G score was low (HD: 12.00, BL: 6.00, *p* = 0.08). Between implant groups, the recall interval was significantly different (*p* = 0.005) and post hoc tests showed significant differences between BL-TiUnite and BL-SIC implants to that respect (BL-TiUnite: 8.85 months, BL-SIC: 11.70 months, *p* = 0.005).

Considering implant-related parameters, significant differences were found for the presence of pus on both implant and group level (*p* = 0.01). However, post hoc tests comparing groups did not reveal significant differences as pus was only observed in the BL-TiUnite group and group-wise comparison was underpowered. Comparison of implant success was statistically significant on implant level (HD: 53.8%, BL: 68.9%, *p* = 0.045) and close to significance on group level (*p* = 0.07). BOP differed significantly on group level (*p* = 0.004) and post hoc tests found again statistically significant differences between BL-TiUnite and BL-SIC implants (BL-TiUnite: 70.4%, BL-SIC: 27.3%, *p* = 0.005). Finally, differences between groups regarding ∆PD failed to be significant by little (*p* = 0.07).

### Assessment of risk factors for – ∆MBL

Multivariate linear regression identified KM and xerostomia as risk factors for – ∆MBL with – ∆MBL being 0.77 units smaller (less bone loss) in average for KM ≥ 1 mm compared to < 1 mm (*p* = 0.0006) and an average increase of 0.02 units in – ∆MBL per additional XI-G score (*p* = 0.09).

### Assessment of risk factors for peri-implantitis

Of all assessed parameters, the risk for peri-implantitis was only significantly enhanced from smokers in comparison to non-smokers (OR 2.87, *p* = 0.013) according to multivariate logistic regression. There was a significant impact of BMI in univariate analysis (OR 0.88 for one additional BMI unit kg/m^2^, *p* = 0.03), but only based on n = 112 observations. A higher plaque score was associated with a risk for peri-implantitis, but failed to reach the significance level (OR 1.36, *p* = 0.07).

### Assessment of risk factors for implant success

Multivariate logistic regression (on success grade 1 = optimum vs grade 2 +  = complications) then identified that more KM (≥ 1 mm vs < 1 mm) was associated with a significantly higher implant success (OR 2.82, *p* = 0.003) and that an additional XI-G score decreased the odds for success (OR 0.95, *p* = 0.007).

## Discussion

### Statement of principal findings

The current study aimed to assess possible differences concerning implant survival and success as well as various parameters of the peri-implant tissues between common implant macro- and micro-design, namely BL and HD implants, for the use in mandibular overdentures. The study revealed within the limits of a retrospective controlled cohort study, that there was no difference concerning the rate of implant failure, or per-implantitis. However, only in one study group there was the presence of peri-implant pus. Implant success according to the Misch criteria was higher in BL-implants with one group being notably higher than the comparing groups, which might have introduced a bias into the results. For bone loss, a width of KM < 1 mm and the presence of xerostomia were identified as risk factors. Smoking and a higher body mass index (BMI) were a risk factor for peri-implantitis. As risk factors for reduced implant success, a small width of KM and the presence of xerostomia were identified.

### Strengths and weaknesses of the study

The current study had some inherent shortcomings, as a retrospective design had to be chosen. Hence, there might have been an inclusion bias, as patients who were very unsatisfied with their previous treatment might have not followed the invitation for the study-related assessment. However, more relevant for the rather low follow-up rate might have been that predominantly old and very old patients who had received the initial implant treatment, and hence, more general factors like reduced mobility, multimobidity, or cognitive impairment might have precluded study participation. In total 80 out of 246 possible study participants agreed to the clinical and radiological follow-up examination, which constitutes a high drop-out rate. First, this might have affected the results directly, and secondly might have led to statistical analyses that might be underpowered for the secondary parameters. A strength was that the sample size calculation was based on the primary outcome parameter “prevalence of peri-implantitis”, and hence, a real conclusion can be drawn out of the current results about this parameter.

Identifying predictors of long-term success and absence of peri-implant disease, the present study extends the evidence to very old patients with results for at least a decade living with a mandibular bar-retained IOD. When interpreting the results, it also must be considered that this retrospective report and investigated parameters were not predefined, medical records of study participants were manually extracted out of the non-digital archive.

Further, it needs to be considered that to avoid statistical fitting problems, implant-related outcomes were not corrected for patients. This means that patients with a higher number of implants are slightly overweighted regarding implant-related outcomes, leading to a small bias for these outcomes.

### Strengths and weaknesses in relation to other studies, discussing particularly any differences in results

Contrary to previous studies, a cause–effect relationship between plaque accumulation and peri-implant mucositis in elderly patients [21] could not be confirmed [21, 22]. A Reason could be the fact that plaque index at time of the follow-up examination does not represent the condition over time. In previous studies, we have found initial clinical evidence, that in elderly patients, peri-implant inflammation might be less severe compared to younger patients and that peri-implant bone loss might be reduced in elderly individuals [23, 24].

Surprisingly, there is very little specific evidence on peri-implant tissue parameters in edentulous patients provided with IODs. Enkling et al. recently published such a report and reported peri-implant bone loss in this indication of − 0.96 ± 0.89 mm. Taking into account, that in the current study, 75% of the implant had been in function between 11 and 15 years, the currently reported peri-implant bone loss seems to be comparably low.

The SLA surface shows a roughness of about Ra = 2–4 μm between the peaks, created by sandblasting with grains of 250–500 μm size followed by an acid bath. Implants with an increased surface “SLActive” contain an enhanced biological activity in comparison with SLA by a purged and directly sealed layer. Under N2 gas protective atmosphere in vials with an isotonic NaCl process, a clean TiO2 passivation layer is created. The specifically SIC invent AG surface treatment method “SIC Matrix” is SLA with zirconia granulate for sandblasting followed by etching and results in a conditioned surface of the implant. The clean SIC Matrix surface achieves a moderate roughness Sa of around 1 µm and a high homogeneity of the topography of the implant surface. A different technique of surface roughening has been applied to “TiUnite” implants that are electrochemically modified by anodic oxidation to increase the thickness of the titanium oxide (TiO2) layer from 17–200 nm in conventional titanium implants to 600–1000 nm. Thus, a porous surface microstructure with pore sizes of about 1.3–2.0 mm^2^ and a moderate degree of surface roughness of Sa = 1 μm is generated [25, 26]. In the current study, peri-implant pus, and implant losses only occurred in implants with the TiUnite surface. Furthermore, BOP-positive implant sites were most frequent in the two TiUnite groups. This is in accordance with results of an animal study, where the TIUnite surface demonstrated enhanced tissue loss in artificial peri-implantitis conditions [27] However, the findings of the present study did not result in differences between the implant types in terms of bone-level changes. A similar outcome was demonstrated in recent evidence, that analyzed bone-level changes among different implant types, including implants with an SLA and TiUnite surface [28–30]. The authors of the review could neither show a significant effect in terms of the implant micro-design between the SLA and TiUnite implants nor of the macro-design within the respective groups, on peri-implant bone-level changes. Consequently, it may be concluded that volume-stable peri-implant conditions can be achieved with all evaluated micro-designs, whereas peri-implant health and survival may be compromised with specific micro-designs [8, 9, 27].

### Meaning of the study: possible mechanisms and implications for clinicians or policymakers

It would be a step backward to interpret no significant association between plaque and disease as an encouragement to forget the best possible oral hygiene in general. Rather, the confidence intervals and missing correlations between plaque and peri-implant diseases contradictory to previous evidence raise the suspicion that still unknown mechanisms underlie the edentulous and elderly patient group [31]. Out of the range of potential explanations, the influence on HD implants, health of the salivary glands as well as the presence of keratinized mucosa around implants might play a key role in peri-implant conditions.

It is interesting to note that a higher BMI was identified as a risk factor for developing peri-implantitis, but this is in line with recent findings from general medicine that may show a link between obesity and inflammation. Ellulu et al. state that adipose tissues release inflammatory mediators and is associated with a multitude of diseases [32]. In this line of thought, a recent review showed a dose–response relationship between the metabolic syndrome and severity and periodontitis [33]. Furthermore, it was demonstrated that obese implant patients show higher BOP, PD and MBL than non-obese controls [34].

### Unanswered questions and future research

In addition to the reduction of risk factors, the establishment of stability factors such as investigation in the relationship between salvia, soft tissue composition and peri-implant health, is a sensible future-oriented investment. Understanding better biological interactions admit modifying risk- and health-stabilizing factors to reduce the potential for disease occurrence or progression.

It can be stated that no validated predictors for long-term peri-implant health exist and no real estimation of factors triggering peri-implant disease can be made. In future reports, applying accurate delineation of the cohort register with consideration of biological conditions is the most important factor to attribute the prevalence of the disease to detect influencing factors.

## Conclusions

For mandibular bar-retained IODs, both bone-level and hybrid-design implants, are mostly successful. A minimum of 1 mm keratinized mucosa around the implants as well as the absence of xerostomia seems to be important for implant success and stable peri-implant bone levels. Besides the well-known risk factor smoking, a high BMI seem to be counterproductive for peri-implant health.

## Data Availability

The datasets used and/or analyzed during the current study are available from the corresponding author on reasonable request.

## Abbreviations

IOD: Implant overdenture
– ∆MBL (mm): Mean peri-implant bone-level changes
HD: Hybrid-design implants, i.e., tissue-level implants with machined neck
HD-SLA: HD with SLA/SLActive surface and machined neck (Institut Straumann, Basel, Switzerland)
HD-TiUnite: HD with TiUnite surface and machined neck (Nobel Biocare AB, Zurich, Switzerland)
BL: Bone-level implants
BL-TiUnite: Bone-level implants with TiUnite surface (Nobel Biocare AB, Zurich, Switzerland)
BL-SIC: BL with SICmatrix surface (SIC invent, Basel, Switzerland)
BOP: Bleeding on probing
PD (mm): Probing depth
∆PD (mm): PD difference between initial value at implant loading and study-related assessment
KM: Keratinized mucosa
XI-G score: Xerostomia inventory (German version)
